# 23.2 NETRIN-1 RECEPTORS CONTROL MESOCORTICAL DOPAMINE CONNECTIVITY IN ADOLESCENCE

**DOI:** 10.1093/schbul/sby014.092

**Published:** 2018-04-01

**Authors:** Cecillia Flores

**Affiliations:** McGill University

## Abstract

**Background:**

Adolescence is an age of heightened vulnerability to develop psychiatric disorders that involve alterations in prefrontal cortex circuitry and cognitive dysfunction. The maturation of prefrontal cortex function is linked to the establishment of dopamine connectivity in this region. Development of mesocortical dopamine is a gradual process that continues until early adulthood. Because of its extended maturational course, this system is particularly susceptible to environmental influences. Yet there is a significant gap in our knowledge about the cellular and molecular mechanisms underlying adolescent prefrontal cortex dopamine development and how they are influenced by experience.

**Methods:**

We examined the role of the Netrin-1 guidance cue receptor, DCC, and its microRNA repressor, miR-218, on adolescent mouse prefrontal cortex development. We used axon-initiated recombination and cell-specific knock-down techniques to characterize the spatiotemporal growth of mesocortical dopamine axons and the role that DCC and miR-218 play in this process. Next, we assessed whether stimulant drugs in adolescence alter miR-218/DCC signaling, thereby disrupting mesocortical dopamine axon growth. Finally, we determined whether altered dopamine axon growth influences prefrontal cortex development by quantifying pyramidal neuron morphology and cognitive performance in adulthood.

**Results:**

Here we show, for the first time, that dopamine axons continue to grow from the nucleus accumbens to the prefrontal cortex during adolescence. We discovered that DCC receptors control the extent of this protracted growth by determining where and when dopamine axons recognize their innervation target. Exposure to stimulant drugs or to stress leads to disruption of DCC-dependent adolescent targeting events, causing dopamine axons that should innervate the nucleus accumbens, to grow ectopically to the prefrontal cortex. This effect profoundly changes prefrontal cortex structural and functional development, producing alterations in cognitive processes known to be impaired across psychiatric conditions, including schizophrenia. Importantly, miR-218 controls DCC receptor expression in dopamine neurons across postnatal development and acts as a molecular mediator of the effects of stimulant drugs on prefrontal cortex development.

**Discussion:**

The prolonged growth of dopamine axons during adolescence represents an extraordinary period for experience to influence their growth and predispose to or protect against psychopathology. MicroRNA control of DCC receptor in dopamine neurons is a molecular link where genetic and environmental factors seem to interact in adolescence to influence the development and function of the prefrontal cortex.

